# Assessment of Prognostic Indicators and Survival-Based Impact of Holistic Approach in Oral Cancer Patients: An Observational Study

**DOI:** 10.7759/cureus.67178

**Published:** 2024-08-19

**Authors:** Sachin S Kandalkar, Manish Sharma, Bhagyashri Ahirrao, Abdul Suban A Kanna, Tauseef A Sheikh, Saudagar M Ziauddin

**Affiliations:** 1 Department of Oral Pathology, Sahkar Maharshi Bhausaheb Thorat Dental College and Hospital, Sangamner, IND; 2 Department of Oral Pathology, Jawahar Medical Foundation's Annasaheb Chudaman Patil Memorial Dental College, Dhule, IND; 3 Department of Pathology, Jawahar Medical Foundation's Annasaheb Chudaman Patil Memorial Medical College, Dhule, IND; 4 Department of Orthodontics, Sri Ramakrishna Dental College and Hospital, Coimbatore, IND; 5 Department of Oral and Maxillofacial Surgery, Aditya Dental College, Beed, IND

**Keywords:** life expectancy, quality of life, survival, squamous cell carcinoma, observational study

## Abstract

Introduction: Oral cancer is recognized as the sixth most common type of cancer globally. Instances have been recorded demonstrating an increase in its incidence, particularly in the territories of southern Asia, with a significant emphasis on India. Thus, the objectives of this investigation were to assess the efficacy of a holistic approach on the life expectancies of patients diagnosed with oral cancer, and to assess the prognostic indicators in such patients.

Material and methods: A retrospective study was conducted on medical records of 60 clinically and histopathologically confirmed cases of oral squamous cell carcinoma (OSCC) who received complete surgical intervention or radiation therapy or a combination of both modalities depending on stage of OSCC from January 2015 to December 2016. After completion of their treatment, 30 patients underwent Cancer Care program of Annabhai Chudamani Patil Memorial Medical College which consisted of yoga sessions, meditation, psychological counselling, nutritional counselling, emotional and social support (embracing a holistic approach, group 1) and 30 patients did not enroll in the Cancer Care initiative (not opting for holistic approach, group 2). The program was conducted for 21 days every six months for two years. Data pertaining to demographic characteristics, stage of OSCC, modalities of treatment administered, histopathological characteristics of the neoplasm, as well as the clinical outcome (Survival/Deceased) post a five-year duration subsequent to the primary diagnosis were extracted from the medical records to assess the role of holistic approach and various factors on the overall survival (OS) of the patients in both the groups. The data collected was subsequently subjected to a thorough statistical analysis.

Results: The mean age of the patients was 44.33±8.66 years (95% CI: 39.53-49.13) in group 1, and 51.20±9.99 years (95% CI: 39.53-49.13) in group 2. The mean survival time for group 1 was 81.60±5.02 months (95% CI: 78.817-84.383), and 66.00±20.29 months (95% CI: 54.761-77.239) in group 2 with statistically significant difference between the groups (p=0.007). Group 2 showed a 1.31 relative risk of mortality to group 1. The probability of death in group 2 was 1.39 times more than in group 1. Cox regression analysis revealed group 2 was significantly associated with the risk of OSCC in this analysis. Other variables were not significantly associated with the risk of the OSCC in this analysis.

Conclusion: The current research indicated that employing a holistic strategy proves to be a successful approach in increasing the OS of patients with OSCC.

## Introduction

Oral squamous cell carcinoma (OSCC) represents a notable global health issue, with prognosis largely influenced by a range of factors, such as the patient's clinical condition, tumour features, and treatment methods. Recently, there has been a call for a holistic strategy for caring for individuals with oral cancer, highlighting the importance of a thorough assessment of the patient's physical, psychological, and social health [1]. OSCC arises within the mucosal epithelium of the oral cavity and represents approximately 90% of oral malignancies. In India, the prevalence of OSCC is markedly more pronounced than in Western countries, with approximately 70% of cases being diagnosed at an advanced stage. This condition adversely affects physical appearance, speech articulation, swallowing function, and the ability to perceive flavours [2]. Treatment of OSCC frequently leads to impairment in function, alteration in appearance, and limitations in physical abilities, all of which have an impact on patients' quality of life (QoL) [3]. The individuals diagnosed with OSCC exhibit a higher prevalence of depressive and anxious symptoms compared to the general population, with psychiatric conditions significantly influencing the prognosis and therapeutic responses in OSCC patients.

In patients with advanced OSCC, there appears to be a notable deficiency in health expectancy, particularly with a prognosis of less than one year, with the exception of individuals aged between 75 and 79 years who received conventional therapeutic interventions [3]. It has been observed that OSCC cell lines actively release interleukin 6 (IL-6), leading to an elevated concentration of this cytokine in both the saliva and blood samples of individuals diagnosed with OSCC leading to significant decrease in overall survival (OS) of such patients. The extent to which different treatment combinations affect the QoL and life expectancy following the acute phase of OSCC has not been extensively documented. Adverse effects commonly associated with traditional radiotherapy for head and neck cancer, with or without concurrent chemotherapy, include radiation-induced skin reactions, inflammation of mucous membranes, nutritional deficiencies, lack of hydration, weight reduction, discomfort, and dry mouth. Long-lasting or postponed negative responses may involve dry mouth, weight loss, voice changes, difficulty swallowing, and hearing impairment [1].

Radiotherapy and surgical interventions for OSCC frequently result in functional and structural impairments. These challenges impede the management of local and regional disease progressions. Regrettably, local and focal recurrences of OSCC remain the primary causes of mortality in affected individuals. The overall recurrence rates range from 21 to 52%. Furthermore, the treatment of OSCC, along with its associated physical morbidity, significantly compromises QoL [4]. Conventional routine medical follow-up frequently does not fulfil the requirement for supportive care. It is common experience to feel deserted during the transition from being a patient to becoming a survivor [5]. Previous research has revealed that individuals diagnosed with stage I, II, or III OSCC exhibit a median survival duration of approximately three to five years, with the highest survival rates observed in Stage 1 and the lowest in Stage 4 [6,7]. Undoubtedly, the majority of Stage III OSCC investigations currently incorporate QoL measures alongside traditional locoregional management and mortality outcomes. Several assessment tools have been developed to evaluate QoL in OSCC, which have received substantial validation and exhibit robust reliability [8].

High oxidative stress has been reported in patients with OSCC [2]. Yoga can significantly affect the duration of cancer remission. Furthermore, yoga exercises may lead to the reversal of epigenetic modifications. Engaging in yoga asanas, meditation, and pranayamas can reduce the risk of carcinogenesis. Maintenance of a robust immune system is essential for exerting anticancer effects in humans. Stress can hinder immunity, thereby promoting the development and progression of cancer [1]. According to the findings of Rao et al., yoga can have positive impacts on the QoL of individuals undergoing cancer treatment. Nonetheless, the research on yoga demonstrates a significant level of diversity in terms of the types of yoga interventions utilized, the duration of the interventions, the level of exposure to yoga practices, and the specific medical indications for which yoga is recommended [9]. The role of holistic approaches, such as yoga, meditation, and emotional and social support, on the life expectancy of patients with OSCC has not yet been studied [9]. Engaging in physical activities among individuals diagnosed with OSCC may lead to enhancements in various aspects such as quality of life, sleep patterns, pain management, depressive symptoms, lean body mass, muscle strength, and physical performance. Substantial evidence supports the notion that physical activity can contribute to prolonged survival rates in patients afflicted with diverse forms of cancer. Therefore, this study aimed to analyse the effects of a holistic approach (yoga sessions, meditation, psychological counselling, nutritional counselling, emotional and social support) on the life expectancy and OS of patients with OSCC. Additionally, the predictors for survival in OSCC patients were also studied. 

## Materials and methods

This retrospective observational study was conducted using the medical records of 60 patients diagnosed with OSCC who underwent complete treatment processes, including surgery, radiotherapy, or combined treatment, between January 2015 and December 2016. After completion of treatment for OSCC, 30 patients in the records participated in the Cancer Care program run by the Annasaheb Chudaman Patil Memorial (ACPM) Medical Foundation, Dhule, Maharashtra for two years, whereas 30 patients refused to enroll in the program. The study was conducted in accordance with the Declaration of Helsinki and approved by the institutional ethical committee (EC/NEW/INST/2022/2959/Y23/035). Written permission was obtained from the ACPM Medical College to access the records of the patients, maintain confidentiality, and use the records for study purposes only. 

Sample size calculation

The sample size was calculated using the G Power software version 3.2.9. The total sample size was calculated at a power of 80%, alpha error of 5%, and 95% confidence interval (CI). The referenced effect size obtained was 1.02 with a mean difference of 15.22 months for survival in OSCC and a standard deviation (SD) of 12.01 between the groups [10]. The estimated sample size was 60 (30 patients per group).

Study design and methodology

Only male subjects with clinically and histopathologically confirmed OSCC who had completed the treatment process as prescribed were included in the study. The presence of hormonal fluctuations in females can introduce variability in stress responses, challenging the generalization of study findings and complicating data interpretation due to the cyclic nature of hormones. Therefore, it was decided to conduct the present study on males only, however, excluding females from studies on stress-relief interventions, such as yoga, can result in gaps in knowledge regarding their specific impacts. Patients with systemic diseases, previous history of irradiation, recurrent OSCC, female subjects, patients who did not receive complete treatment of OSCC, and patients with OSCC as a secondary malignancy were excluded from the study.

All the selected cases were divided into two groups: 30 case records (with a holistic approach, group 1) where the Cancer Care program was given, and 30 case records (without a holistic approach, group 2) where the Cancer Care program was not given. The Cancer Care program provided yoga, meditation (spiritual), psychological counselling, nutritional counselling, and emotional and social support after the completion of treatment for OSCC. The Cancer Care program was functional for 21 days every six months. A program lasting 42 days in a year guaranteed that individuals were consistently assisted and monitored. This facilitated the early identification of potential complications or adverse reactions, enabling prompt interventions. The remaining 323 days within the calendar year afforded a significant period for recuperation, adjustment to any changes in lifestyle, and continuous post-treatment support. This prolonged duration played a vital role in the management of adverse effects, modification of therapies, and surveillance for any signs of relapse. In essence, although a 42-day program might be intensive, the subsequent 323 days provided room for adaptability in care, recovery, and adjustment, presenting a well-rounded strategy for cancer management throughout the year. The yoga sessions were conducted four times weekly for 45 minutes each, involving moderate-intensity exercises emphasizing gentle postures, breathing techniques, and relaxation methods. The meditation activities were carried out on a daily basis lasting 20 minutes each, with a primary focus on enhancing mindfulness, promoting relaxation, and alleviating stress. Counselling sessions were conducted on a weekly basis, ranging from 45 minutes to one hour in duration, to address psychological concerns. Nutritional guidance was offered every other week for 30 minutes per session, tailored to individual dietary needs. Weekly group sessions lasting 45 minutes to an hour were organized to offer emotional and social support.

**Figure 1:**
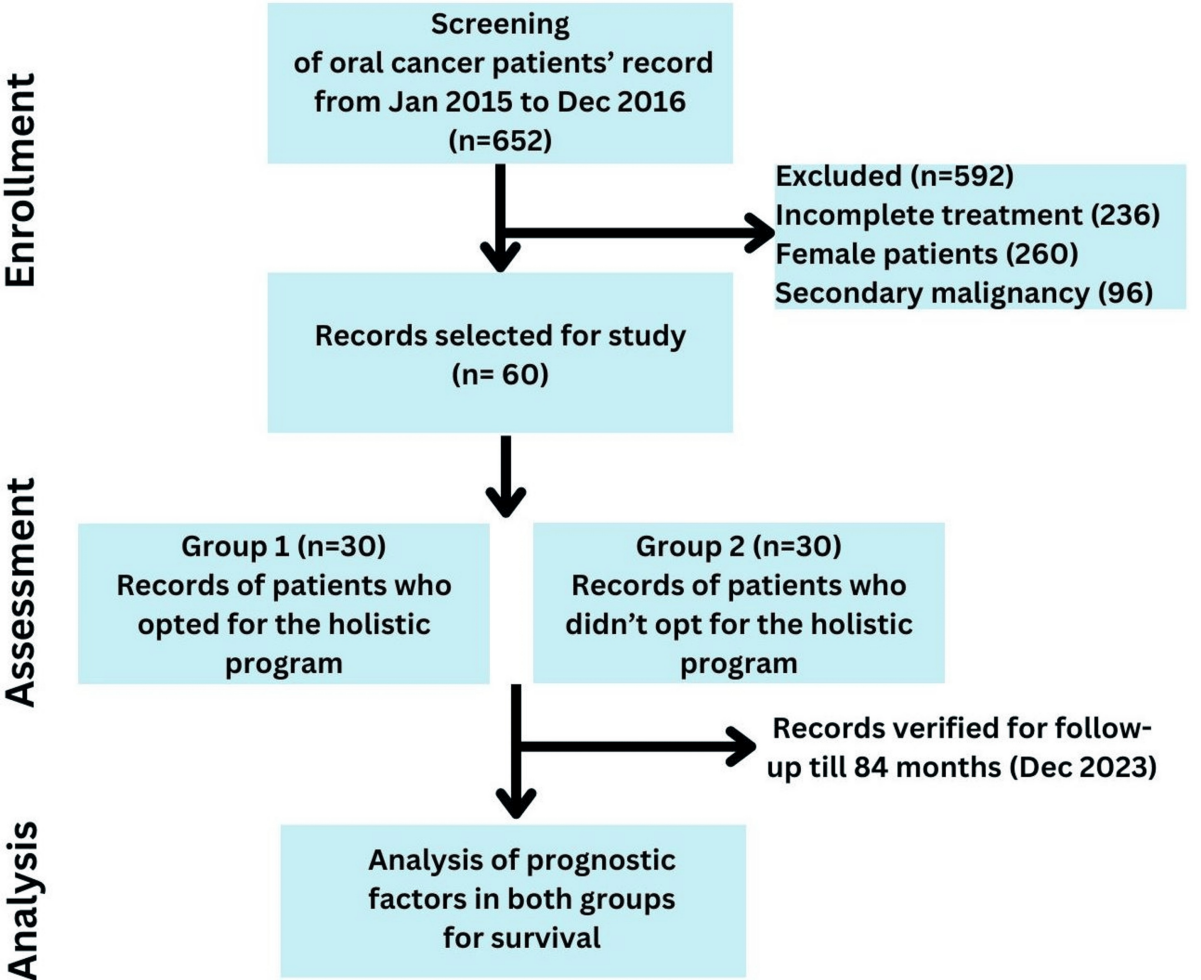
Study design

Group 1 consisted of medical records of the patients who attended the program for two years. All cases were followed up regularly: every month for two years, once every three months for the third and fourth years, and then once every six months for life. The initial two years post-cancer therapy are of utmost importance due to the fact that the likelihood of cancer reoccurrence is typically highest within this timeframe. Subsequent to this period, the immediate risk of reoccurrence diminishes, leading to a reduction in the frequency of follow-up appointments to once every three months. Moving past the fourth year, the probability of reoccurrence continues to decrease; however, maintaining a long-term follow-up schedule of every six months throughout one's life is essential in ensuring timely detection of any delayed reoccurrences or secondary malignancies. There were no instances of attrition in the study, given its retrospective nature, and only data from patients who conscientiously completed the seven-year follow-up period were included. The duration of OS was defined as the time from the moment of diagnosis to the occurrence of death or the date of the final follow-up, whichever came first. December 31, 2023, was established as the deadline for documenting the ultimate follow-up (84 months of follow-up). Data pertaining to demographic characteristics, stage of OSCC, site and size of lesion, modalities of treatment administered, histopathological characteristics of the neoplasm, as well as the clinical outcome (survival/deceased) after a seven-year period subsequent to the primary diagnosis were extracted from the medical records. Patient life expectancy was assessed in both groups (Figure 1). The anonymity of the patient was preserved during data analysis and reporting by following measures: By de-identifying the data which involved assigning a distinct code or identifier to each patient's data, ensuring the anonymity of personal information. The data was presented in a summarized manner, for instance through averages or percentages, rather than on an individual basis. The primary researcher had exclusive access to the original data, while the data analyst worked with the anonymized, coded data. Rigorous scrutiny of this publication was conducted to prevent the inclusion of any potentially identifiable information.

Statistical analysis

Statistical analyses were performed using IBM Statistical Package for Social Sciences (SPSS) version 23 (IBM Corp., Armonk, NY, USA). The obtained data were sent for descriptive analysis to study significant changes in the lifespan of patients after holistic treatment by the Cancer Care program at 95% CI. Baseline clinical and pathological variables (quantitative data) are presented as frequencies, percentages, mean and SD. The focal point was the survival aspect, known as OS, meticulously calculated using the Kaplan-Meier product-limit technique. The Kaplan-Meier method was employed to estimate OS within different patient cohorts for OS, survival by age, site, recurrence, stage of tumour differentiation, type of treatment, and lymph node metastasis. Univariate analysis of OS was performed, focusing on captivating clinical and histopathological factors carefully chosen from previous investigations of oral cavity cancer: age group, primary site, habits (tobacco smoking, tobacco chewing, and/or alcohol consumption), tumour differentiation, lymph node metastasis, tumour size, treatment, and recurrence. A multivariate Cox proportional hazards regression analysis was performed to evaluate the influence of established significant prognostic elements, as multivariate analysis would allow researchers to study the relationship between multiple factors affecting OS of OSCC patients. The independent samples t-test was used for parametric data and the chi-square test of association was used for non-parametric data. All p-values below 0.05 were deemed to indicate statistical significance.

## Results

Sixty patients who were operated between January 2015 to December 2016 were included in the present study according to the eligibility criteria. Table 1 depicts the descriptive characteristics of the study sample.

**Table 1:** Descriptive characteristics of study group OSCC: Oral squamous cell carcinoma; Data presented in n (%) form

Descriptive characteristics	Group 1 n(%)	Group 2 n(%)	Total n(%)
Age group (years)			
<45	18 (60.00%)	10 (33.33%)	28 (46.67%)
>45	12 (40.00%)	20 (66.67%)	32 53.33%)
Habit			
Tobacco	22 (73.33%)	22 (73.33%)	44 (73.33%)
Tobacco+Alcohol	8 (26.37%)	8 (26.37%)	16 (26.67%)
Primary Site			
Buccal Mucosa	24 (80.00%)	14 (46.66%)	38 (63.33%)
Floor of the mouth	0 (0.00%)	8 (26.37%)	8 (13.37%)
Gingiva	6 (20.00%)	8 (26.37%)	14 (23.33%)
OSCC differentiation			
Moderate	12 (40.00%)	10 (33.33%)	22 (36.67%)
Poor	8 (26.37%)	8 (26.37%)	16 (26.66%)
Well	10 (33.33%)	12 (40.00%)	22 (36.67%)
Tumours size			
2-4 cm	16 (53.33%)	8 (26.37%)	24 (40.00%)
<2cm	2 (6.67%)	10 (33.33%)	12 (20.00%)
>4cm	12 (40.00%)	12 (40.00%)	24 (40.00%)
Lymph node metastasis			
Absent	22 (73.33%)	24 (80.00%)	46 (76.67%)
Present	8 (26.37%)	6 (20.00%)	14 (23.33%)
Bone invasion			
Absent	22 (73.33%)	20 (66.67%)	42 (70.00%)
Present	8 (26.37%)	10 (33.33%)	18 (30.00%)
Treatment			
Surgery	8 (26.37%)	10 (33.33%)	18 (30.00%)
Surgery+Radiotherapy	14 (46.66%)	14 (46.66%)	28 (46.67%)
Surgery+Radiotherapy+Chemotherapy	8 (26.37%)	6 (20.00%)	14 (23.33%)
Recurrence			
No	24 (80.00%)	20 (66.67%)	44 (73.33%)
Yes	6 (20.00%)	10 (33.33%)	16 (26.67%)

The majority of patients in group 1 who survived (n=12, 33.34%) and those who passed away (n=6, 25%) were under 45 years of age. In contrast, in group 2, the numbers of surviving patients (n=8, 22.22%) were equal for age below or above 45 years, while most deceased patients (n=12, 50%) were in this age group of more than 45 years. There was no statistically significant difference among the surviving patients (p=0.549), but a statistically significant difference was observed among the deceased patients (p=0.019). The prevalent habit among surviving patients was tobacco use, whereas deceased patients in group 2 commonly used both tobacco and alcohol. The most affected site was the buccal mucosa in group 1 (n=16, 44.44%) and in group 2 (n=8, 22.22%) among survivors, followed by the gingiva in group 1 (n=4, 11.12%) and in group 2 (n=8, 22.22%). Interestingly, the floor of the mouth was not affected in deceased patients of group 1, but this site was involved in eight (33.33%) deceased patients in group 2, showing a statistically significant difference between the two groups (p=0.006). The deceased patients exhibited tumour sizes exceeding 4 cms in six patients (25%) within group 1 and 12 patients (50%) in group 2. Patients in group 2 who underwent a combination of surgery and radiotherapy (Type 2) had higher mortality rates, while those in group 1 who received surgery, radiotherapy, and chemotherapy (Type 3) experienced more deaths. Over a seven-year follow-up, the deceased patients with recurrences were evenly distributed between the two groups. Group 2 demonstrated a greater recurrence rate (n=6, 16.66%) compared to group 1 (n=2, 5.56%) among surviving patients, indicating that a comprehensive approach significantly reduced recurrence in OSCC patients. However, once OSCC recurred, it did not prevent the deaths of those individuals. Bone invasion was more common in deceased patients in both groups, although the disparity was not statistically significant (p=0.633). Most tumours in surviving patients from both groups were moderately or well-differentiated during the follow-up period, while poorly differentiated OSCC was the primary cause of death in both groups. Lymph node metastasis was present in eight patients (33.33%) in group 1 and six patients (25.00%) in group 2 among deceased patients, with no statistically significant variance (p=0.07) (Table 2).

**Table 2:** Descriptive characteristics of the study groups and association between groups analyzed by chi-square test of association *Fisher exact test, **p<0.05 significant Data presented in n (%) form OSCC: Oral squamous cell carcinoma, Type 1: Surgery, Type 2: Surgery and radiotherapy, Type 3: Surgery, radiotherapy, chemotherapy

Patient during follow-up period	Variables	Group		p-value
		Group 1	Group 2	
	Age group (years)			
Survived	<45	12 (33.34%)	8 (22.22%)	0.549
	>45	8 (22.22%)	8 (22.22%)	
Deceased	<45	6 (25.00%)	2 (08.33%)	0.019**
	>45	4 (16.67%)	12 (50.00%)	
	Habit			
Survived	Tobacco	16 (44.44%)	16 (44.44%)	0.058
	Tobacco+Alcohol	4 (11.12%)	0 (00.00%)	
Deceased	Tobacco	6 (25.00%)	6 (25.00%)	0.408
	Tobacco+Alcohol	4 (16.67%)	8 (33.33%)	
	Primary Site			
Survived*	Buccal Mucosa	16(44.44%)	8(22.22%)	0.081
	Floor of the mouth	0 (00.00%)	0 (00.00%)	
	Gingiva	4 (11.12%)	8 (22.22%)	
Deceased	Buccal Mucosa	8 (33.33%)	6 (25.00%)	0.006**
	Floor of the mouth	0 (00.00%)	8(33.33%)	
	Gingiva	2 (08.34%)	0 (00.00%)	
	OSCC differentiation			
Survived*	Moderate	10 (27.78%)	4 (11.12%)	0.176
	Poor	0 (00.00%)	0 (00.00%)	
	Well	10 (27.78%)	12 (33.34%)	
Deceased*	Moderate	2 (08.34%)	6 (25.00%)	0.388
	Poor	8 (33.33%)	8 (33.33%)	
	Well	0 (00.00%)	0 (00.00%)	
	Tumour size			
Survived	2-4 cm	12 (33.34%)	6 (16.67%)	0.001**
	<2cm	2 (5.56%)	10 (27.78%)	
	>4cm	6 (16.67%)	0 (00.00%)	
Deceased*	2-4 cm	4 (16.67%)	2 (08.34%)	0.192
	<2cm	0 (00.00%)	0 (00.00%)	
	>4cm	6 (25.00%)	12(50.00%)	
	Lymph node metastasis			
Survived*	Absent	20 (55.56%)	16 (44.44%)	0.000**
	Present	0 (00.00%)	0 (00.00%)	
Deceased	Absent	2 (08.34%)	8 (33.33%)	0.069
	Present	8 (33.33%)	6 (25.00%)	
	Bone invasion			
Survived*	Absent	20 (55.56%)	16 (44.44%)	0.000**
	Present	0 (00.00%)	0 (00.00%)	
Deceased	Absent	2 (08.34%)	4 (16.67%)	0.633
	Present	8 (33.33%)	10 (41.66%)	
	Type of treatment			
Survived*	Type 1	8 (22.22%)	10 (27.78%)	0.315
	Type 2	12 (33.33%)	6 (16.67%)	
	Type 3	0 (00.00%)	0 (00.00%)	
Deceased*	Type 1	0 (00.00%)	0 (00.00%)	0.104
	Type 2	2 (08.34%)	8 (33.33%)	
	Type 3	8 (33.33%)	6(25.00%)	
	Recurrence			
Survived	No	18 (50.00%)	10 (27.78%)	0.049**
	Yes	2 (5.56%)	6 (16.66%)	
Deceased	No	6 (25.00%)	10 (41.66%)	0.558
	Yes	4 (16.67%)	4(16.67%)	

During 84 months of follow-up, 36 patients (60.00%) survived, whereas 24 patients (40.00%) passed away (deceased) with no statistically significant difference between the groups (p=0.291). In group 1, 10 patients (16.67%) passed away (deceased) within an 84-month follow-up period, while in group 2, 14 patients (23.33%) deceased during the same tracking period. Group 2 showed a 1.31 relative risk of mortality to group 1. The probability of death in group 2 was 1.39 times more than in group 1, as shown in Table 3.

**Table 3:** Comparison of death during follow-up between groups *chi square test, p<0.05 significant Data presented in n(%) form

Patient during follow-up period	Group 1	Group 2	Total	P value*	Relative risk	Odd ratio	Probability
Survived	20 (33.33%)	16 (26.67%)	36 (60.00%)	0.291	1.31	1.75	1.39
Deceased	10 (16.67%)	14 (23.33%)	24 (40.00%)				

**Figure 2:**
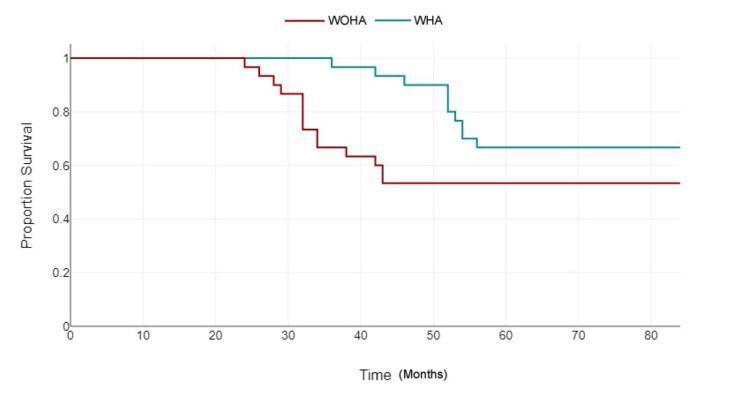
Kaplan Meier survival plot for the study groups WOHA: Without holistic approach; WHA: With holistic approach

The mean survival time for group 1 was 81.60±5.02 months (95% CI: 78.817-84.383), and 66.00±20.29 months in group 2 (95% CI: 54.761-77.239) with a statistically significant difference between the groups (p=0.007), as shown in Figure 2.

The mean tumour size was 3.79±1.42cms (95% CI: 3.004-4.582) in group 1, and 3.60±1.57cms (95% CI: 2.726-4.474) in group 2 with no statistically significant difference between the groups (p=0.727). The mean age of the patients was 44.33±8.66 years (95% CI: 39.53-49.13) in group 1, and 51.20±9.99 years (95% CI: 39.53-49.13) in group 2 with no statistically significant difference between the groups (p=0.052), as shown in Table 4.

**Table 4:** Independent sample t-test analysis to compare study groups in relation to survival period, tumor size and age factor *p<0.05 significant, SD: Standard deviation, CI: Confidence interval Data presented in form of mean±SD

Parameters	Group	N	Mean±SD	95% CI		Minimum	Maximum	p value
				Upper	Lower			
Survival period (months)	Group 1	30	81.60±5.02	84.38	78.81	68	84	0.007*
	Group 2	30	66.00±20.29	77.23	54.76	34	84	
Tumour size (cm)	Group 1	30	3.79±1.42	4.58	3.00	1.8	6.5	0.727
	Group 2	30	3.60±1.57	4.47	2.72	1.5	6.4	
Age (years)	Group 1	30	44.33±8.66	49.13	39.53	34	62	0.052
	Group 2	30	51.20±9.99	56.73	45.66	34	65	

**Figure 3:**
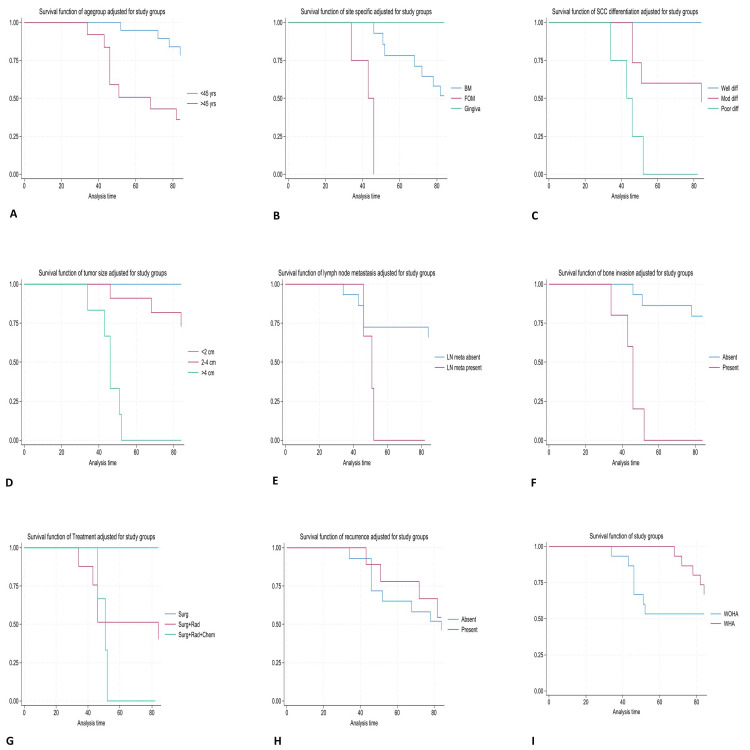
Kaplan-Meier survival curves adjusted for study groups. A. Survival curve for age groups; B. Survival curve for site of tumour; C. Survival curve for oral squamous cell carcinoma (OSCC) differentiation; D. Survival curve for tumour size; E. Survival curve for lymph node metastasis; F. Survival curve for underlying bone invasion; G. Survival curve for type of treatment; H. Survival curve for recurrence of OSCC; I: Survival curve for groups

Kaplan Meier survival analysis (Table 5) adjusted for age group revealed that patients aged above 45 years have survival of 57.33 months, whereas all the patients below 45 years of age survived for 74 months (Figure 3A).

**Table 5:** Comparison of survival characteristics by Kaplan Meier analysis OSCC: Oral squamous cell carcinoma

Variables	Category	Time at risk (days)	Incidence rate	Average survival period	Survival time (months)		
					25%	50%	75%
Group	Group 1	2177	0.004	74.00	54	84	84
	Group 2	1813	0.007	66.67	32	84	84
Age group	>45 years	1945	0.008	57.33	34	54	84
	<45 years	2045	0.003	74.00	54	84	84
Primary Site	Buccal mucosa	2556	0.005	70.33	43	84	84
	Floor of mouth	319	0.025	36.33	32	34	43
	Gingiva	1115	0.001	84.00	84	84	84
Habit	Tobacco	3163	0.003	74.00	54	84	84
	Tobacco+Alcohol	827	0.014	43.67	34	43	54
Tumour size	<2cm	1008	0.000	84.00	84	84	84
	2-4cm	1757	0.003	74.00	54	84	84
	>4cm	1225	0.140	43.67	32	43	56
OSCC differentiation	Well	1848	0.000	84.00	84	84	84
	Moderate	1495	0.005	49.67	22	43	84
	Poor	647	0.024	40.00	32	36	52
Lymph node metastasis	Absent	3461	0.002	84.00	84	84	84
	Present	529	0.026	35.67	29	32	46
Bone invasion	Absent	3267	0.001	84.00	84	84	84
	Present	723	0.024	40.67	32	38	52
Type of treatment	Type 1	1512	0.000	84.00	84	84	84
	Type 2	1949	0.005	73.33	52	84	84
	Type 3	529	0.026	35.67	29	32	46
Recurrence	Absent	2934	0.004	73.33	52	84	84
	Present	1056	0.009	56.00	32	52	84

The patients who had OSCC at floor of the mouth had the lowest survival of 36.33 months, followed by buccal mucosa of 70.33 months. All the patients who had OSCC of gingiva survived for 84 months (Figure 3B). The patients having well-differentiated OSCC had higher survival of 84 months than poorly differentiated OSCC (40 months) (Figure 3C). Patients with tumour size more than 4 cm have short survival of 43.67 months (Figure 3D), with lymph node metastasis had a survival of 35.67 months (Figure 3E), and with bone invasion had a survival of 40.67 months (Figure 3F). Patients who had undergone treatment with Type 3 treatment had the shortest survival of 35.7 months, followed by 73.33 months survival with type 2 treatment, and all the patients with type 1 treatment survived for 84 months (Figure 3G). Patients with recurrence of OSCC had a survival of 56 months, compared to 73.33 months in patients without recurrence (Figure 3H). Group 1 had a longer average OS of 74 months than group 2 of 66.67 months (Figure 3I).

Cox regression hazard analysis revealed that there was a statistically significant difference in hazard ratios between study groups. Individuals in group 2 had a hazard ratio of 0.04 (95% CI: 0.006 to 0.28) compared to those in group 1, and this difference was statistically significant (p=0.001). The results suggested that group 2 was significantly associated with the risk of the event in this analysis. Other variables were not significantly associated with the risk of the event in this analysis, as depicted in Table 6.

**Table 6:** Cox regression hazard analysis for multiple variables *p<0.05 significant OSCC: Oral squamous cell carcinoma

Variables	Category	Hazard ratio	p value	95% Confidence interval	
				Lower	Upper
Group	Group 1	0.04	0.001*	0.00	0.28
	Group 2				
Age group	<45 years	1.02	0.979	0.19	5.24
	>45 years				
Primary Site	Buccal mucosa	0.57	0.479	0.12	2.60
	Floor of mouth				
	Gingiva				
Habit	Tobacco	2.76	0.369	0.30	25.50
	Tobacco+Alcohol				
Tumour size	<2cm	0.47	0.490	0.05	3.90
	2-4cm				
	>4cm				
OSCC differentiation	Well	2.69	0.180	0.63	11.42
	Moderate				
	Poor				
Lymph node metastasis	Absent	1.03	0.968	0.19	5.39
	Present				
Bone invasion	Absent	0.33	0.300	0.04	2.64
	Present				
Type of treatment	Type 1	2.30	0.651	0.61	8.03
	Type 2				
	Type 3				
Recurrence	Absent	0.88	0.833	0.27	2.85
	Present				

## Discussion

Oral cancer is a condition that affects the entirety of an individual, encompassing not only the head and neck region. Individuals encountering a diagnosis of oral cavity cancer and the ensuing treatment encounter notable challenges [11]. Our study aimed to explore the life expectancy of patients who received holistic treatment after oral cancer treatment. Though there are several studies on the Western population that have reported OS and quality of life after OSCC, however, none of them have assessed the effects of holistic approach on OS of patients with OSCC and the factors affecting OS in such patients [5,6,8,9,11]. The concept of "survivorship experience" embodies a comprehensive narrative of the cancer patient's life journey, incorporating elements related to the patient's physical health, emotional wellness, standard of living, and interpersonal relationships [10]. The most common priorities for patients' preferences are cure of disease, improved QoL, and trust in healthcare providers.

The findings of the current investigation demonstrated that individuals diagnosed with OSCC who are older than 45 years, have OSCC located in the floor of the mouth, possess tumours larger than 4 cm with infiltration of the underlying bone, and exhibit lymph node metastasis along with poorly differentiated OSCC managed through a multimodal approach involving surgery, radiotherapy, and chemotherapy, were linked to a decreased OS. These findings were in agreement with previous studies [12-14]. These findings indicate that forecasting the outcome of patients with OSCC is a complex task, necessitating the inclusion of many factors in the prognostic evaluation. Contrarily it has been reported that lower survival rates were observed in younger individuals [15]. Even the location of OSCC did not appear to affect the survival of OSCC patients [16]. Additional investigation is warranted in light of the incongruent data pertaining to survival rates based on gender and age. Poorly differentiated neoplasms exhibit a correlation with heightened rates of recurrence among individuals diagnosed with OSCC, thus warranting vigilant monitoring and potential use of supplementary treatment modalities in these particular instances. Alcohol and tobacco use are known risk factors for oral cancer.

The present study revealed that holistic approach improved the life expectancy of the patients, and increased the OS time. This finding was in agreement with a previous study [17]. The group in which patients did not opt for holistic approach after OSCC treatment showed 1.31 relative risk of occurring death than the group who opted for holistic approach. The probability of death in group 2 was 1.39 times more than in group 1. Spirituality is a consequence that has garnered relatively limited focus compared to other outcomes. The provision of spiritual care constitutes a specific domain within the field of nursing, facilitating favourable encounters for both healthcare providers and recipients. Extensive scholarly examination has been dedicated to this particular notion, revealing that spirituality constitutes a fundamental element of individual identity and an intrinsic occurrence intertwined with personal virtuosity, cognition, and benevolence. This notion is distinguished by a belief in a divine entity or a superior force and interpersonal engagement [18].

The reason for improved OS in patients of OSCC with holistic approach could be due to the fact that yoga and exercises lower the levels of IL-6, which has been acknowledged as a cytokine that holds significance in the processes of angiogenesis and tumoral advancement in OSCC [19]. Prior research has demonstrated that individuals diagnosed with oral cancer may experience elevated levels of psychological distress [20,21]. In a research conducted by Kugaya et al. [22], findings indicated that among 107 individuals examined, 18 patients (16.8%) exhibited symptoms of either adjustment disorder or major depression. It is recommended that patients with OSCC presenting advanced stages of the disease or those living in solitude undergo thorough evaluation to identify psychological distress and enable appropriate intervention strategies. Bernabe and colleagues [22] have documented that stress among patients with OSCC resulted in an upregulation of IL-6 production in reaction to stress, consequently contributing to the progression of stress-related OSCC. The increased prevalence of depressive symptoms in OSCC warrants use of holistic approach in such patients [21]. The influence of neurohormonal mediators, stemming from prolonged stress, on the modulation of oral cancer cell behaviour has been documented [23]. Research findings have underscored the involvement of neurohormones in facilitating the invasive and progressive tendencies across various cancer types through beta-adrenergic pathways [9,23,24]. Moreover, the elevation in norepinephrine (NE) levels within the tumour microenvironment has been identified as a predictive factor for the development of OSCC [24].

The results of our study supported the notion that physical activity can contribute to prolonged survival rates in patients afflicted with diverse forms of cancer. The associations between physical activity and heightened overall survival rates have been reported in the studies in comparison to individuals leading a sedentary lifestyle [25,26]. The investigation conducted by Bakshi and colleagues delved into the impact of yoga therapy on the quality of life of individuals afflicted with oral cancer. Their findings led to the conclusion that the incorporation of yoga alongside standard treatments in a comprehensive approach has the potential to enhance the quality of life for these patients [27].

The utilization of an integrative care strategy in our study, which takes into account non-pharmacological methods like yoga, meditation, psychological and nutritional counselling led to better OS and improved QoL for the patients with OSCC who opted for holistic approach, as noticed in a previous study also [28]. The notion of social support comprises various facets, such as informational, emotional, self-esteem, tangible, and social elements. Individuals experience a sense of gratitude when they receive social support, perceiving that they are valued and embraced by others. The existence of encouraging interpersonal connections carries the potential to impact the well-being of individuals who have survived cancer. Survivors of cancer necessitate social support from their loved ones and companions to attain optimal health. Mehta et al. in a review highlighted the benefits of mediation in cancer management [29]. Koshimoto et al. concluded that multidisciplinary assistance pertaining to nutritional manifestations, as well as psychological elements such as anxiety and confusion, and social factors like household composition and employment status, could potentially augment the quality of life for cancer patients receiving outpatient chemotherapy [30]. 

Our research findings presented a notable rise in the mean survival duration by 15.6 months among OSCC individuals employing a holistic strategy. Those patients who declined the holistic approach displayed elevated rates of disease recurrence and mortality. These results highlight the substantial impact of embracing a holistic approach on enhancing overall survival and diminishing recurrence rates in OSCC patients. Cox regression analysis also suggested that holistic approach was the important factor in explaining the differences in the survival time between the groups. The present study was conducted in male patients only as it has been documented in previous studies that stress responses differ significantly in males and females [31]. The key strengths of the study were long follow-up period of seven years, first study to evaluate the role of holistic approach on OSCC, and detailed study of various factors influencing the OS of OSCC patients in both groups.

The key findings of our research revealed that the consumption of both tobacco and alcohol leads to a decrease in OS in patients with OSCC. The presence of poorly differentiated OSCC in the buccal mucosa and the floor of the mouth necessitates a comprehensive treatment approach involving surgery, radiotherapy, and chemotherapy, highlighting the importance of early detection and management of OSCC. Implementing stress management strategies in these individuals has the potential to enhance their QoL and OS.

Limitations

The major drawback of the study was its retrospective nature, which limits the large amount of information required for the study such as any previous history of using stress reduction protocols, detailed history of habits, and data about immunohistochemical analysis. The small sample size was another drawback for the present study. To eliminate bias due to gender-based differences in stress response, only male subjects were included in the study. The stress response was not evaluated in the present study due to the retrospective nature of the study. Though the follow-up of the patients was seven years in the present study, however, future randomized control trials with longer follow-up periods are required to assess the role of holistic approach in OSCC patients of both genders.

## Conclusions

The article highlights the importance of the holistic approach in increasing the life expectancy of oral cancer patients after treatment by analyzing the critical components related to long-term survival. Holistic approach led to a significant increase in overall survival time of the oral cancer patients. The patients aged more than 45 years, poorly differentiated squamous cell carcinoma, involvement of underlying bone and lymph node metastasis, history of tobacco and alcohol consumption, lesions involving floor of the mouth, and patients who underwent combination of surgery, radiotherapy and chemotherapy were the predictors for poor prognosis in oral cancer patients.
